# Self-injurious behaviour in patients with bipolar disorder and attention deficit hyperactivity disorder after central stimulant start– a retrospective study based on the lisie cohort

**DOI:** 10.1192/j.eurpsy.2021.239

**Published:** 2021-08-13

**Authors:** L. Öhlund, M. Ott, R. Lundqvist, M. Sandlund, E. Salander Renberg, U. Werneke

**Affiliations:** 1 Sunderby Research Unit, Department Of Clinical Sciences, Division Of Psychiatry, Umeå University, Umeå, Sweden; 2 Department Of Public Health And Clinical Medicine, Division Of Medicine, Umeå University, Umeå, Sweden; 3 Sunderby Research Unit, Department Of Public Health And Clinical Medicine, Umeå University, Umeå, Sweden; 4 Department Of Clinical Sciences, Division Of Psychiatry, Umeå University, Umeå, Sweden

**Keywords:** attention deficit disorder with hyperactivity, central nervous system stimulants, self-injurious behaviour, bipolar disorder

## Abstract

**Introduction:**

Currently, our understanding remains limited of how co-occurring bipolar disorder and attention deficit hyperactivity disorder (ADHD) should be treated.

**Objectives:**

To evaluate the impact of central stimulant treatment on self-injurious behaviour in patients with a dual diagnosis of bipolar disorder or schizoaffective disorder and ADHD.

**Methods:**

Retrospective cohort study (LiSIE) into effects and side-effects of lithium as compared to other mood stabilisers. Here, using a mirror-image design, we compared suicide attempts and non-suicidal self-injury events within 6 months and 2 years before and after central stimulant treatment start.

**Results:**

Of 1564 eligible patients, 206 patients met inclusion criteria; having a dual diagnosis of bipolar disorder or schizoaffective disorder and ADHD at first central stimulant initiation. In these, suicide attempts and non-suicidal self-injury events decreased significantly within both 6 months (p = 0.004) and 2 years (p = 0.028) after central stimulant start. After multiple adjustments, this effect was preserved 2 years after central stimulant start (OR 0.63, 95% CI; 0.40 – 0.98, p = 0.041).

**Conclusions:**

Central stimulant treatment may reduce the risk of self-injurious behavior in patients with a dual diagnosis of bipolar disorder or schizoaffective disorder and ADHD. However, to reduce the risk of manic switches, concomitant mood stabilising treatment remains warranted.

**Disclosure:**

Michael Ott has been a scientific advisory board member of Astra Zeneca Sweden, Ursula Werneke has received funding for educational activities on behalf of Norrbotten Region (Masterclass Psychiatry Programme 2014–2018 and EAPM 2016, Luleå, Sweden): Astra

